# Transfusion of Red Blood Cells to Patients with Sepsis

**DOI:** 10.3390/ijms18091946

**Published:** 2017-09-11

**Authors:** Yi-Ling Chan, Shih-Tsung Han, Chih-Huang Li, Chin-Chieh Wu, Kuan-Fu Chen

**Affiliations:** 1 Department of Emergency Medicine, Chang Gung Memorial Hospital Linkou, Taoyuan 330, Taiwan; ylchan@cgmh.org.tw (Y.-L.C.); adrianhst@gmail.com (S.-T.H.); chhli2002@gmail.com (C.-H.L.); 2 Department of Emergency Medicine, Chang Gung Memorial Hospital Keelung, Keelung 204, Taiwan; wujinja@cgmh.org.tw; 3 Clinical Informatics and Medical Statistics Research Center, Chang Gung University, Taoyuan 330, Taiwan; 4 Community Medicine Research Center, Chang Gung Memorial Hospital Keelung, Keelung 204, Taiwan

**Keywords:** sepsis, transfusion, red blood cells

## Abstract

Sepsis is one of the major causes of death worldwide, and is the host response to infection which renders our organs malfunctioning. Insufficient tissue perfusion and oxygen delivery have been implicated in the pathogenesis of sepsis-related organ dysfunction, making transfusion of packed red blood cells (pRBCs) a reasonable treatment modality. However, clinical trials have generated controversial results. Even the notion that transfused pRBCs increase the oxygen-carrying capacity of blood has been challenged. Meanwhile, during sepsis, the ability of our tissues to utilize oxygen may also be reduced, and the increased blood concentrations of lactate may be the results of strong inflammation and excessive catecholamine release, rather than impaired cell respiration. Leukodepleted pRBCs more consistently demonstrated improvement in microcirculation, and the increase in blood viscosity brought about by pRBC transfusion helps maintain functional capillary density. A restrictive strategy of pRBC transfusion is recommended in treating septic patients.

## 1. Introduction

Sepsis claims more than 200,000 lives annually in the United States [1]—more than prostate cancer, breast cancer, and acquired immunodeficiency syndrome combined [2]. Sepsis is the major cause of death for patients in the intensive care unit [3]. Packed red blood cell (pRBC) transfusion has been incorporated into the recommended treatment bundle of sepsis since 2004 [4]. This review discusses the rationale of pRBC transfusion during sepsis, its efficacy, and concerns that this practice has generated.

## 2. Sepsis

The terms “pyremia”, “toxemia”, or “septicemia” have been used to denote the disease state induced by bacterial infections. Patients often present with fever, shaking chills, and rapid heartbeat. Severely ill patients may also have drowsiness, rapid breathing, sweating, decreased urine output, and low blood pressure. In 1980, with the discovery of tumor necrosis factor (TNF) alpha and the characterization of its pathogenetic role in endotoxin poisoning [5], clinicians postulated that the disease state of toxemia is actually the response of our body (i.e., the “host response”) to the invading bacteria. The nature of the response is the mobilization of the immune system to powerfully eradicate the bacteria, thus it is overwhelming, severe, and “inflammatory”. Such a response seems inducible not only by bacterial infections, but also by other infective microorganisms (e.g., viral, fungal, or malarial infections) as well as several non-infectious etiologies (e.g., trauma, burns, and pancreatitis). This response—later termed the “systemic inflammatory response” [6]—is not entirely dangerous, but when it becomes dysregulated and uncontained, it can damage our own tissue, cause the malfunctioning of our organs, and kill the patient. In 1991, the American College of Chest Physicians and the Society of Critical Care Medicine held a consensus conference, and defined “sepsis” as the systemic inflammatory response syndrome induced by infection [6]. Severe sepsis denotes sepsis accompanied by organ dysfunction, and septic shock denotes sepsis-induced hypotension that cannot be reversed by intravenous fluid resuscitation.

The immunopathogenesis of sepsis has been reviewed elsewhere [7]. In brief, certain structures of the infective microorganisms are “recognizable” by several groups of surface receptors on our cells. The structures are referred to as the pathogen-associated molecular patterns, and the corresponding surface receptors are referred to as the pattern recognition receptors [8]. Toll-like receptors (TLRs) are among the four families of the pattern recognition receptors [9]. Of the 11 members of TLR in mammals, TLR1, 2, 4, and 6 recognize bacterial lipids; TLR 9 recognizes bacterial deoxyribonucleic acids, and TLR5 and 10 recognize bacterial or parasite proteins. TLR signaling pathways activate several transcription factors, which subsequently induce the generation and release of several kinds of cytokines, chemokines, and interferons from the cell. The upstream proinflammatory cytokines—including TNF alpha and interleukin 1 beta—further activate the downstream cytokines, lipid mediators, reactive oxygen species, cell adhesion molecules, coagulation cascades, and endothelium. Certain physiological abnormalities ensue, including the hypercoagulable state [10], the excessive generation of nitric oxide leading to vascular relaxation and formation of reactive nitrogen species [11], and shedding of endothelial glycocalyx leading to increased capillary leak [12]. Intravascular thrombosis and vasodilation cause tissue hypoperfusion, and excessive generation of reactive oxygen and nitrogen species causes cell disruption, increased apoptosis [13], and mitochondrial injuries [14].

Conventional treatment for sepsis includes the eradication of the invading microorganisms through the prompt use of antimicrobial agents, identification and control of the infection focus and the causative pathogen, support of dysfunctional organs, and maintenance of tissue perfusion during hypotension. However, regarding the overwhelming inflammation, therapies including high-dose steroids [15] and anti-TNF antibody [16] failed to improve survival. Nevertheless, in the late 1990s, several studies demonstrated results that seemed promising in the treatment of sepsis. These included the use of recombinant human activated protein C [17], low-dose hydrocortisone [18], tight glycemic control [19], and early goal-directed therapy (EGDT) [20]. These positive results have prompted the Surviving Sepsis Campaign [21], which aimed to lower sepsis mortality by 25% over five years [22].

However, and quite frustratingly, subsequent studies failed to confirm the beneficial effects of these positive studies [23,24,25,26,27,28]. One of the reasons to account for this divergence could be the lack of specificity of sepsis criteria. The original definition of sepsis relies on abnormal changes in two or more among several clinical parameters, as well as the presence of infection. However, the change in the clinical parameters in question is actually quite non-specific, as it can be induced by many non-inflammatory conditions. Moreover, at the time of patient inclusion into a study, the diagnosis of infection was often based on clinical suspicion. About half of the patients diagnosed with severe sepsis could end up having negative culture results [29]. Consequently, clinical studies might have enrolled patients whose symptoms were not actually induced by infection or inflammation. The lack of response in these patients to a particular treatment could have diluted the true beneficial effect of the therapy [30].

In light of this, the Society of Critical Care Medicine and the European Society of Intensive Care Medicine have revised the consensus definitions for sepsis (designated to as “Sepsis-3”) and septic shock in 2016 [31]. Sepsis is now defined as “life-threatening organ dysfunction caused by a dysregulated host response to infection”. Septic shock is now defined as presence of hypotension and hyperlactatemia (increased blood concentrations of lactate) in the absence of hypovolemia.

## 3. Rationale for Packed Red Blood Cell (pRBC) Transfusion during Sepsis

**Figure 1:**
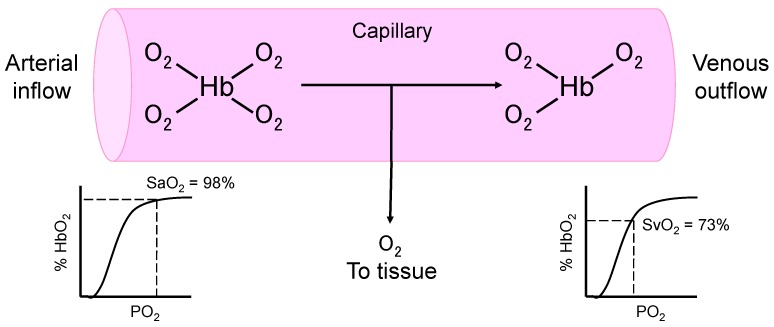
The delivery of oxygen molecules by the hemoglobin (Hb) to the peripheral tissue. Depending on the oxygen tension of the tissue, one or several oxygen molecules will be released from the oxyhemoglobin (HbO_2_) and diffuse from the capillary (pink area) into the tissue. The fraction of oxyhemoglobin relative to total hemoglobin in arterial blood is the arterial oxygen saturation (SaO_2_, as percentage), which is normally >95%. Normal mixed venous oxygen saturation (SvO_2_) is about 65–75%. A decrease in SvO_2_ usually indicates low tissue oxygen tension, resulting in increased extraction of oxygen molecules from the hemoglobin. Figure adapted from [35].

Septic patients with hypotension and hyperlactatemia carry a high mortality [32]. Hyperlactatemia has long been presumed to be the result of circulatory collapse and tissue hypoperfusion, leading to cellular hypoxia, disrupted mitochondrial respiration, and lactic acid fermentation. The presence of “oxygen debt” is reflected by the decrease of mixed venous oxygen saturation [33], which signifies the increased extraction of oxygen molecules from the hemoglobin under low tissue oxygen tension (Figure 1). Thus, for patients with hypotension, hyperlactatemia, and decreased mixed venous oxygen saturation (normal values, 70–80% [34]), conventional treatment includes volume expansion to optimize cardiac output, vasopressors to maintain adequate perfusion pressure, and an increase in the oxygen carrying capacity of the blood to allow more oxygen molecules to be transported to the peripheral tissue. In theory, pRBC transfusion can increase hemoglobin concentrations, and thereby increase the oxygen carrying capacity of the blood (Table 1).

In the 1990s, several clinical studies evaluated the effect of achieving supranormal oxygen delivery (>600 mL/min/m^2^) on mortality in high-risk patients. Supranormal oxygen delivery could be achieved by increasing blood hemoglobin levels and/or by increasing cardiac output with inotropes like dobutamine. Shoemaker et al. reported that in a cohort of 253 high-risk surgical patients, those who died or developed organ failure had higher estimated oxygen debt [37]. In a separate cohort of 56 high-risk surgical patients, 27 received protocolized treatment to achieve a supranormal cardiac output and oxygen delivery. These patients ended up having reduced oxygen debt, fewer organ failures, and lower mortality [37]. Yu et al. conducted a randomized controlled trial on 67 critically ill patients and found that patients who achieved high oxygen delivery—either with treatment or self-generated—did present with a markedly lower mortality [38]. In contrast, Hayes et al. recruited 109 critically ill patients and found that achieving supranormal oxygen delivery actually increased the in-hospital mortality [39]. When looking specifically at septic patients in this cohort, they found a similar trend as Yu et al., that survivors could be characterized by the ability to increase oxygen delivery and consumption [36]. Gattinoni et al. found no differences in mortality in critically ill patients receiving specific treatment to increase cardiac output or to normalize mixed venous oxygen saturation [40].

Finally, Rivers et al. reported that increasing oxygen delivery in the early phase of treatment could improve mortality in septic patients with hypotension or hyperlactatemia [20]. All patients received standard therapy including intravenous volume expansion and vasopressors to restore normal blood pressure. Patients in the EGDT group further received pRBC transfusion to achieve a hematocrit of ≥30%, and intravenous dobutamine infusion to achieve a central venous oxygen saturation of ≥70%, within 6 h after emergency department admission. In this study, the average baseline central venous oxygen saturation was below 50%, suggesting the presence of increased oxygen extraction from the upstream tissues. At the 6-hour timepoint, 95% of patients in the EGDT group had a central venous oxygen saturation of ≥70%, compared with only 60% of patients in the standard-therapy group. pRBCs were transfused in 64% of patients in the EGDT group and 19% in the standard-therapy group. The authors highlighted the importance of correcting oxygen debt in the early phase so that subsequent pathophysiological abnormalities could have been prevented from happening. In addition, Park et al. reviewed 1054 patients with severe sepsis and septic shock. They reported that after propensity-score matching, patients receiving pRBC transfusion had lower 28-day and in-hospital mortality risks [41].

## 4. Have Transfused RBCs Increased Oxygen Delivery?

As EGDT seemed promising in lowering mortality in septic patients with shock or hyperlactatemia, many questioned the necessity of pRBC transfusion to increase oxygen delivery. In healthy humans, tissue oxygen consumption can be maintained even at a hemoglobin level of 5 g/dL [42]. That is, normal circulation possesses a great reserve of oxygen delivery. Given this fact, why would severely septic patients present with hyperlactatemia and decreased mixed/central venous oxygen saturation? Two plausible explanations are: (1) during sepsis, the circulatory function may be altered, leading to a decreased reserve in oxygen delivery; and/or (2) the pathophysiology underlying hyperlactatemia and decreased mixed/central venous oxygen saturation is not totally oxygen debt.

Accumulating data indicated that oxygen delivery remains sufficient during sepsis. A phenomenon termed “pathological supply dependency” of tissue oxygen consumption during sepsis has been described [43]. The “supply dependency” means that the tissue requires more oxygen than normal circulation can provide. The reserve of oxygen delivery is no longer sufficient, making pRBC transfusion a reasonable choice to increase the oxygen-carrying capacity of the blood flow. However, subsequent studies have invalidated this “supply dependency” [44,45]. Specifically, the systemic oxygen consumption in septic patients is not dependent on oxygen delivery, even in the presence of hyperlactatemia [46]. In fact, few sepsis studies have reported a presenting mean central venous oxygen saturation of <70% [47].

**Figure 2:**
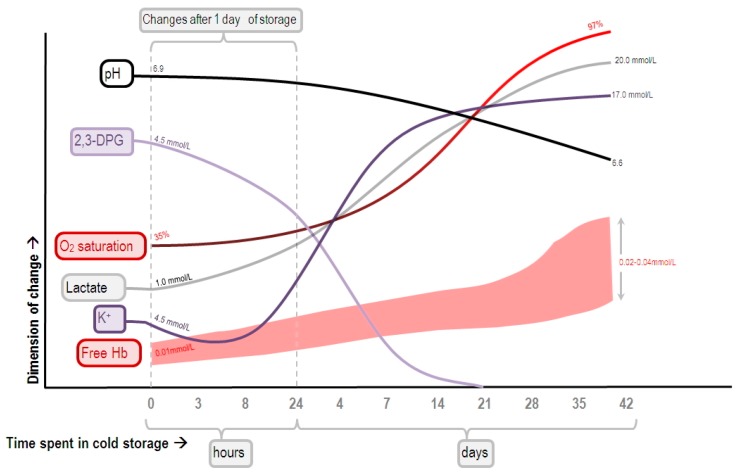
Schematic representation of the changes of pH, and the concentrations of 2,3-diphosphoglycerate (2,3-DPG), lactate, potassium, and free hemoglobin (Hb) in stored red blood cells. Figure adapted from [49] by Alex Yartsev. Reproduced with permission from Alex Yartsev, Deranged Physiology website (http://www.derangedphysiology.com), accessed on 29 May 2017.

In addition, the increase in mixed/central venous oxygen saturation after pRBC transfusion actually does not necessarily signify the correction of oxygen debt. Ince et al. showed that during hypovolemia and sepsis, the capillary oxygen tension might be lower than the venous oxygen tension [48], indicating the presence of shunting from the microcirculation to the venous compartment. This could mean that the ability of the microvasculature to regulate blood flow according to the tissue’s need has diminished, and that tissue hypoxia may persist despite the mixed/central venous oxygen saturation has been restored to normal values. Meanwhile, the oxygen affinity of the hemoglobin molecule varies with the concentration of 2,3-diphosphoglycerate (2,3-DPG; higher levels favor oxygen release). In banked pRBCs, 2,3-DPG is rapidly depleted (Figure 2) [49]; its level will not return to normal until 6 to 12 h after being transfused [50], and sometimes it takes as long as 72 h [51]. This results in a decreased release of oxygen molecules from the transfused RBC, and a subsequent increase in venous oxygen saturation. Thus, the increase in central venous oxygen saturation at the 6-hour timepoint in patients receiving pRBC transfusion might have resulted not from the correction of oxygen debt, but from shunting and/or the increased oxygen affinity of the transfused RBC.

Moreover, it has been speculated that pRBC transfusion may paradoxically hamper oxygen delivery. RBCs can change their shape to pass through capillaries smaller than their diameters. This “deformability” of RBCs was thought to greatly influence the blood viscosity and flow resistance in the small blood vessels [52]. RBC deformability is decreased during sepsis [53,54,55]. The deformability of banked pRBCs is also decreased for several reasons [56]. These include a change in RBC membrane property, the depletion of 2,3-DPG and adenosine triphosphate (ATP), loss and internalization of membrane phospholipids, loss of *S*-nitrosothiols, protein rearrangement, and lipid oxidation [57,58] (see ref. [59] for an updated review of RBC storage lesions). Animal experiments demonstrated that transfusion of RBCs rendered less deformable caused a decrease in blood flow and microvascular density [55]. However, some authors argued that the experimental models might have exaggerated the effect that stored RBC could have caused, claiming that less deformable RBCs would simply pass through vessels of larger diameters [60]. On the other hand, clinical studies showed inconsistent results. Marik et al. measured systemic oxygen uptake using indirect calorimetry in 23 critically ill septic patients, and found that pRBC transfusion did not increase systemic oxygen uptake [61]. They also found that patients receiving pRBCs stored >15 days constantly developed a decrease in gastric intramucosal pH, suggestive of decreased tissue oxygenation. However, this finding was not reproduced by a later study [62]. We compared patients with severe sepsis who either received or did not receive pRBC transfusion during the first day in the emergency department. After matching 177 pairs of patients using propensity score generated by patient demographics, comorbidities, organs dysfunction, and severity of sepsis, we found no difference in the risk of in-hospital mortality [63]. Sadaka et al. measured tissue oxygen saturation using near-infrared spectroscopy and found that in severely septic patients with anemia, lactic acidosis, or a central venous oxygen saturation of <70%, pRBC transfusion did not alter muscle oxygen consumption or microvascular reactivity. However, pRBC transfusion improved muscle oxygen consumption in patients with altered baseline levels, but paradoxically deteriorated muscle oxygen consumption in patients with preserved baseline levels [64]. This disparity again reflects the heterogeneity in characteristics of the enrolled patients. Using a microdialysis catheter inserted into the subcutaneous adipose tissue, Kopterides et al. found that pRBC transfusion decreased the interstitial lactate/pyruvate ratio in critically ill septic patients [65]. The authors also noted marked inter-individual variations of the observed changes, and patients who had the greatest decrease in lactate/pyruvate ratio after transfusion tended to be those who had the highest ratio before transfusion.

If it is possible that stored pRBCs negatively impact oxygen delivery, would fresh pRBCs circumvent this disadvantage? No studies have specifically addressed the effect of fresh pRBC transfusion in septic patients. However, in a prospective randomized controlled trial on critically ill patients, fresh pRBC transfusion showed no difference from stored pRBCs in terms of mortality risks, duration of organ support, or hospital lengths of stay [66]. On the other hand, a small prospective randomized trial in 20 septic patients receiving either leukodepleted (i.e., pRBCs from which the white blood cells have been removed) or non-leukodepleted RBCs showed no difference in tissue oxygenation, but patients receiving leukodepleted RBCs more consistently demonstrated improvement in microcirculation [67].

Importantly, Tsai et al. demonstrated that the maintenance of microvascular function relies not only on the oxygen-carrying capacity or *S*-nitrosothiol content of RBCs, but also on adequate blood viscosity, which maintains functional capillary density [68]. In a hamster model of hemorrhagic shock, animals receiving carbon monoxide- or nitrate-treated RBCs showed no difference in systemic and microcirculatory hemodynamics [69,70]. Using a mathematical model, Zimmerman et al. estimated the effect of an increase in blood viscosity on oxygen delivery. Their model predicted that with baseline hemoglobin concentrations of >5.8 g/dL, transfusion of 0.5–3.0 units of pRBCs would increase blood viscosity, which subsequently led to a decrease in oxygen delivery [71]. Therefore, pRBC transfusion may ultimately help maintain microcirculation by restoring blood viscosity. This effect appears to be independent of the oxygen carrying capacity. A similar effect is hopefully attainable in the future by using plasma expanders that increases plasma viscosity (e.g., polyethylene glycol-conjugated albumin) [72].

Regarding the decision threshold of pRBC transfusion (the “transfusion trigger”) in septic patients, a large prospective randomized controlled trial compared the hemoglobin levels of 7 and 9 g/dL in patients with septic shock. The TRISS trial found no difference in 90-day mortality, ischemic events, or use of life support between the two transfusion triggers [73]. A meta-analysis that included 12 cohort studies of critically ill septic patients also found that the restrictive pRBC transfusion strategy (using the hemoglobin level of 7 g/dL as the transfusion trigger) was not associated with increased mortality in critically ill septic patients [74].

## 5. Does the Tissue Need More Oxygen during Sepsis?

The idea that our tissues actually need more oxygen during sepsis has also been challenged. Brealey et al. reported the decreased activity of the mitochondrial respiratory chain in the skeletal muscle of septic patients [75]. The excessive generation of nitric oxide during sepsis may inhibit complexes I and IV of the mitochondrial respiratory chain through nitrosylation and nitration [14]. Inhibition of complex IV can render the mitochondria incapable of utilizing oxygen to produce ATP [76]. Indeed, a retrospective study found that in patients with septic shock, the median central venous oxygen saturation within the first 72 h was higher in nonsurvivors [77]. However, the levels of central venous oxygen saturation were widely distributed, equally apart in survivors and nonsurvivors. This again demonstrated the markedly heterogeneous presentation of septic patients. Dyson et al. [78] measured tissue oxygen tension in a rodent model, and found that hypoxemia induced a generalized decrease in tissue oxygen tension, whereas progressive hemorrhage caused declines of oxygen tension in liver, muscle, and urinary bladder, but not in renal cortex. In contrast, endotoxemia caused a rise in bladder and a fall in liver oxygen tension, but muscle and renal cortical oxygen tension remained unchanged. Thus, inter-organ differences in oxygen delivery and utilization exist. Other studies have demonstrated parallel but variable results (reviewed by Arulkumaran et al. [79]), which may be attributable to the model used, the tissue analyzed, and the timing of measurement. The extent to which the mitochondrial dysfunction contributes to sepsis-related organ dysfunction remains uncertain [79]. Singer speculated that mitochondrial inhibition during sepsis could be a process of hibernation, to avoid the excessive generation of reactive oxygen/nitrogen species that cause tissue damage [76].

Moreover, during sepsis, hyperlactatemia may not necessarily indicate the presence of oxygen debt. James et al. found that skeletal muscle production of lactate could be related to the activity of plasmalemmal Na^+^-K^+^-ATPase pump, and muscles from septic animals had an increased rate of lactate production [80]. Levy et al. showed that in patients with septic shock, the concentrations of lactate and pyruvate in quadriceps muscles were consistently higher than the arterial blood. However, this gap can be eliminated by ouabain (the inhibitor of Na^+^-K^+^-ATPase) [81]. The authors speculated that there is a process of accelerated glycolysis in the skeletal muscle, inducible by catecholamine and inflammation. This led to the excessive generation of pyruvate and lactate, irrespective of the status of tissue oxygenation. Hyperlactatemia was present in healthy volunteers receiving bacterial endotoxin injection, while tissue oxygen saturation remained normal [82]. Therefore, hyperlactatemia may signify the presence of severe diseases, but it does not necessarily represent the existence of oxygen debt, nor does it spontaneously justify the prescription of pRBC transfusion.

## 6. Complications of pRBC Transfusion during Sepsis

pRBC transfusion can cause complications ranging from allergic reactions, transfusion-related infections, and febrile reactions, to more severe complications like acute lung injury, circulatory overload, immunomodulation, or hemolysis [83]. In an active surveillance in California that involved 463,207 units of blood and blood components, the incidence of transfusion-related acute lung injury in 2009 was only 0.81 per 10,000 units transfused [84]. Even though the risk is low, it is still an unnecessary risk for patients not likely to benefit from pRBC transfusion (e.g., those with no evidence of defected oxygen delivery). Moreover, a meta-analysis of 12 cohort studies revealed that pRBC transfusion in critically ill septic patients was associated with the occurrence of nosocomial infection, acute lung injury, and acute kidney injury [74]. Patients receiving RBCs of longer shelf lives had a higher incidence of infection, and critically ill patients receiving older RBCs had a higher mortality risk [85]. Transfusion of stored RBCs increased the blood level of non-transferrin-bound iron [86]. Sera with higher levels of non-transferrin-bound iron have been shown to enhance the proliferation of several kinds of bacteria in vitro [86,87]. It is uncertain whether this phenomenon has been implicated in the increased incidence of infection in patients receiving older RBCs. Nevertheless, it is recommended that pRBC transfusion should be used judiciously in patients with sepsis [88].

## 7. Conclusions

Sepsis is the disease state caused by the dysregulated and uncontained inflammatory response to infection. However, the term “sepsis” does not simply describe a homogeneous disease state, but the whole spectrum of a very wide ranges of presentations: from hyper-inflammation to immunoparesis, from early to later phase, from focusing on infection to inflammation, and from killing bacteria to modulating immunity. It is therefore advised that one needs to specify “what do you mean by sepsis?” to set the context. Conventional treatment focuses on infection control and organ support, but currently not much can be done to modulate immune responses. pRBC transfusion appears to be helpful to some patients with sepsis—especially those “presented” with defective oxygen delivery. However, the presentation itself may vary widely and may well be misleading, and pRBC transfusion does not guarantee an increase in oxygen delivery. A restrictive strategy of pRBC transfusion is therefore recommended in treating septic patients. Mitochondrial dysfunction may underlie sepsis-related organ dysfunction, but further studies are required to fully elucidate its role.

## Figures and Tables

**Table 1:** Hemodynamic parameters.

Cardiac index (L/min/m^2^) = Cardiac output/body surface area
Arterial oxygen content (mL/dL) = 1.39 × Hb (g/dL) × SaO_2_ + 0.0225 × PaO_2_ (kPa)
Mixed venous oxygen content (mL/dL) = 1.39 × Hb (g/dL) × SvO_2_ + 00225 × PvO_2_ (kPa)
C(a–v)O_2_ = arterial oxygen content − mixed venous oxygen content
DO_2_ (mL/min/m^2^) = cardiac index × arterial oxygen content × 10
VO_2_ (mL/min/m^2^) = cardiac index × C(a–v)O_2_ × 10
Oxygen extraction ratio = C(a–v)O_2_/arterial oxygen content

C(a–v)O_2_: arteriovenous oxygen difference; DO_2_: oxygen delivery; Hb: hemoglobin; PaO_2_: arterial oxygen partial pressure; PvO_2_: mixed venous oxygen tension; SaO_2_: arterial oxygen saturation; SvO_2_: mixed venous oxygen saturation; VO_2_: oxygen consumption. Adapted from [36].

## Abbreviations

2,3-DPG	2,3-Diphosphoglycerate
ATP	Adenosine triphosphate
C(a–v)O_2_	Arteriovenous oxygen difference
DO_2_	Oxygen delivery
EGDT	Early goal-directed therapy
Hb	Hemoglobin
HbO_2_	Oxyhemoglobin
PaO_2_	Arterial oxygen partial pressure
pRBCs	Packed red blood cells
PvO_2_	Mixed venous oxygen tension
SaO_2_	Arterial oxygen saturation
SvO_2_	Mixed venous oxygen saturation
TLR	Toll-like receptors
TNF	Tumor necrosis factor
VO_2_	Oxygen consumption
